# Distinct Cecal and Fecal Microbiome Responses to Stress Are Accompanied by Sex- and Diet-Dependent Changes in Behavior and Gut Serotonin

**DOI:** 10.3389/fnins.2022.827343

**Published:** 2022-04-12

**Authors:** Joshua M. Lyte, Lucas R. Koester, Karrie M. Daniels, Mark Lyte

**Affiliations:** ^1^Poultry Production and Product Safety Research, Agricultural Research Service, United States Department of Agriculture, Fayetteville, AR, United States; ^2^Department of Veterinary Microbiology and Preventive Medicine, College of Veterinary Medicine, Iowa State University, Ames, IA, United States

**Keywords:** microbial endocrinology, microbiota-gut-brain axis, diet, chronic stress, behavior, serotonin, gut

## Abstract

Although diet- and stress-induced perturbations in the microbiome (biotic and abiotic factors) associate with changes in host behavior via the microbiota-gut-brain axis, few mechanisms have been identified. The identification of causative pathways by which the microbiome influences host behavior therefore would benefit from the application of evidence-based conceptual frameworks. One such causal framework is microbial endocrinology which is the study of neuroendocrine axes as avenues of bi-directional neurochemical-based host-microbe crosstalk. As such, we investigated the relationship between diet- and stress-induced alterations in behavior, regional gut serotonergic response, and concomitant changes in the cecal and fecal bacterial populations of male and female mice. Our results demonstrate that sex is a dominant factor in determining compositional changes in the gut microbiome in response to stress and diet modifications. Intestinal serotonergic responses to stress were observed in both sexes but dietary modifications uniquely affected region-specific changes in males and females. Likewise, behavioral alterations diverged between male and female mice. Together, these results demonstrate distinct sex-dependent relationships between cecal and fecal bacterial taxa and behavioral- and serotonergic-responses to stress and diet. The present study demonstrates the importance of including both male and female sexes in the examination of the microbiota-gut-brain axis. As different microbial taxa were identified to associate with the behavioral and gut serotonergic responses of male and female mice, certain bacterial species may hold sex-dependent functional relevance for the host. Future investigations seeking to develop microbiome-based strategies to afford host stress resilience should include sex-based differences in the microbiome.

## Introduction

Patterns of association between the gut microbiome and psychosocial outcomes in animals and humans have led to the proposal that the microbiota-gut-brain axis is relevant in the design of dietary strategies seeking to improve stress management as well as, broadly, mental health (for reviews see Ratsika et al., 2021). Like many scientific fields which utilize animal models, microbiome research does suffer from a bias toward the use of male animals instead of both sexes. This is particularly problematic as the areas of mental health in which the microbiome has been proposed as a treatable target, have been reported to unevenly afflict men and women (Rubinow and Schmidt, 2019), as well as diverge among male and female animals (Kokras and Dalla, 2014). Similarly, differences in the composition of the gut microbiome have been reported between the sexes (Org et al., 2016; Bridgewater et al., 2017), and early life stress has been reported to differently impact the microbiota of male and female mice (Rincel et al., 2019). Yet, few studies have investigated whether sex-based differences in the microbiome are related to established mechanisms of the microbiota-gut-brain axis that are implicated in stress susceptibility and behavioral outcomes relevant to mental health.

The field of microbial endocrinology, which is the intersection of neurobiology and microbiology, has demonstrated that neurochemical-based host-microbe crosstalk serves as a causal route of the microbiota-gut-brain axis (Lyte, 2014). The synthesis of several neurochemicals, including stress-related catecholamines, does occur in the gut and is a process that has been demonstrated to be both affected by stress and partly dependent on the microbiome (Asano et al., 2012). Dietary interventions reported to affect host behavior were also found to affect gut neurochemical metabolism (van de Wouw et al., 2020). Likewise, diet was shown to have a sex-dependent effect on the gut microbiota (Bolnick et al., 2014; Zhang et al., 2020). While the majority of microbial endocrinology-based investigations have employed male animals (Li et al., 2009) and have found translational success into the clinical literature (Lyte and Bailey, 1997), little has been done to examine whether sex-based differences in the microbiome determine an effect of dietary intervention on host behavior through altered gut neurochemical response to stress.

Although Metchnikoff proposed that lactic acid bacteria found in yogurt may aid in the improvement of mood (Bested et al., 2013), it was not until the last two decades that dietary manipulation of the gut microbiome and resulting changes in taxa diversity were proposed to be linked to changes in host behavior (Li et al., 2009; Lyte, 2013; Selhub et al., 2014; Goyal et al., 2015). We had previously identified an association between diet-induced changes in the gut microbiome and working memory in male mice fed a diet composed of 50% lean beef compared to mice fed a chow-only diet. Amino acids, such as tryptophan, found in beef are precursors for stress-related neurochemicals including serotonin and have been demonstrated to mediate physiological and cognitive resilience to stress (Hulsken et al., 2013). Oral intake of tryptophan has also been reported in men and women to affect working memory under stressful conditions (Firk and Markus, 2009; Strasser et al., 2016). Probiotic species, as well as other bacterial species found in the gut have been reported to utilize diet-derived amino acids to synthesize neurochemicals (Villageliu and Lyte, 2018). As enterochromaffin cells sample luminal contents (Bellono et al., 2017), luminal concentrations of serotonin may affect gut-to-brain communication via EEC paracrine activation of vagal afferents (Zhu et al., 2001). However, no study to date has investigated whether sex differences in microbiome response to stress co-occur with alterations in gut serotonin concentrations following stress, as well as associate with host resilience in memory and learning.

While several studies have tied diet-induced changes in the fecal bacterial communities to alterations in host behavior (Li et al., 2009), the composition and function of the microbiome and therefore its relevance to the host varies according to anatomical gut region. Likewise, vagal innervation and serotonin concentrations differ along the biogeography of the gut (Beaver and Wostmann, 1962; Bonaz et al., 2018). For example, the interaction of host sex and the microbiome was recently demonstrated to play an important role in determining gut serotonergic response to stress in a region-dependent manner (Lyte et al., 2020). Microbial endocrinology-based circuits of the microbiota-gut-brain axis, then, may be influenced by host sex and should not be assumed to be universal throughout the entire length of the gut but instead to have some degree of anatomical specificity, especially considering neuronal, immunological, and morphological characteristics that distinguish different regions of the gastrointestinal tract. It is well understood that the degree of innervation by the enteric nervous system varies greatly along the intestinal tract with anatomical regions in the small intestine having greater innervation and integration with the vagus nerve that communicates to the central nervous system and brain than portions of the large intestine (Furness et al., 2014). Thus, an understanding of biogeography is crucial to elucidating mechanistic pathways by which host neurobiology and microbiology can interact and ultimately influence behavior. Further, despite recent evidence suggesting sex differences in the fecal gut microbiota may influence behavioral responses in male and female rodents (Bridgewater et al., 2017), little attention has been directed toward whether sex differences that might occur in other gut regions, such as the cecum, are also relevant to sexual divergent host behavior.

As such, we utilized male and female mice to investigate the role of sex in determining the relationship between diet- and stress-induced changes in the gastrointestinal bacterial communities, gut serotonin concentrations, and host behavior. As our previous work showed a diet of 50% beef compared to a chow-only diet improved memory and learning in male mice, an effect that was associated with compositional changes in the microbiome, we employed the same diet here but in male and female mice. Likewise, models of chronic unpredictable stress have been demonstrated to uniquely affect male and female mice behavior, with distinct changes in the gut microbiome. In the present study, we have employed the approach of quantitating the regional neurochemical biogeography during stress coupled with an examination of microbial diversity to infer microbial endocrinology-based mechanistic pathways by which interaction between these two components could determine behavioral outcome in males and females as measured in standard behavioral tests. Hence, we hypothesized that microbial endocrinology-based relationships between diet, stress, and behavior would diverge based on host sex.

## Materials and Methods

### Ethical Approval

All procedures were approved by the Iowa State University Institutional Animal Care and Use Committee (IACUC-18-329) before the study was initiated.

### Animals

Male and female CR-1 mice (Charles River, Wilmington, MA) were acquired at 5 weeks of age. Upon arrival, mice were randomly allocated into experimental groups and housed in standard wire-top cages at a density of 2 same-sex mice per cage. Male and female experimental groups (*n* = 9–10 mice per sex/group) consisted of (1) Control (chow only) diet, (2) Control (chow only) diet + stress, (3) Beef-chow mix diet, and (4) Beef-chow mix diet + stress. Mice were allowed to acclimate to cage conditions while being maintained on standard pellet chow diet for a period of 2 days at which point mice in the beef-chow mix diet group were permanently switched to the beef-chow mix diet. All groups were maintained under normal conditions until 7 weeks of age at which time the stress and behavioral components of the present study were started. For the entire duration of the study, mice were maintained under a reverse 12 h light/12 h dark cycle and provided feed and water *ad libitum*. Estrous cycle stage in female mice was not monitored in the present study. Bodyweight was monitored and recorded weekly.

### Diet

Mice in the chow and chow only + stress groups were maintained on a ground diet that contained no animal source of protein (2019S ground diet, Envigo Teklad Diets, Madison, WI). A ground diet was used to approximate the same consistency of feed as the beef-chow mix diet. The beef-chow mix and beef-chow mix + stress groups were provided a 50% raw ground beef/50% Envigo 2019S (w/w) diet. The same batch of Envigo 2019S diet was used in the diets of all groups. The beef-chow mix diet required mashing of beef and chow, and therefore was not suitable for standard overhead feeding in wire-top cages. As such, the beef-chow mix diet was fed to mice in a small ceramic container that was situated on the bedding of each cage. To ensure consistency of texture and feeding method, mice in the control diet groups received the ground diet in an identical small ceramic container on the cage floor. All food, and ceramic food containers, in each group were monitored and replaced daily with freshly prepared food and clean containers between 12:00 and 17:00. Extra-lean USDA-certified whole cut beef was purchased from a local supermarket, and within the laboratory, processed into ground beef. Microbiological plating of the ground beef was conducted to confirm absence of bacterial contamination before feeding to mice.

### Experimental Design

At 7 weeks of age, mice in the stress groups were subjected each day to either restraint or forced swim stress. All stress procedures were performed during the animals’ active cycle. The daily alternation of restraint and forced swim stressors continued until mice were sacrificed. Immediately, or one day after, the commencement of the stress paradigm, mice of each group begun a 4 day period of Barnes maze training. Barnes maze training or testing each day occurred before exposure to that day’s stressor. Barnes maze testing was conducted on Day 1 and then again at 1 week following the training session. All mice were tested a single time on the elevated plus maze (EPM) 1 day before sacrifice. All mice were sacrificed via bilateral thoracotomy at 10 weeks of age. The same researcher performed all behavioral tests in order to minimize any inter-individual differences in handling or other effects on the mice (Sorge et al., 2014).

### Restraint Stress

The restraint device consisted of a custom-made perforated plexiglass device (Zamora-Gonzalez et al., 2013). The restraint device was kept in a separate room that served as a dedicated space for stress sessions. To perform the restraint stress, cages were transported to the room in which the restraint device was housed. The mouse was allowed to acclimate in its cage for 30 min in the restraint room. After 30 min, the mouse was gently placed into the restraint device. The total period of time for each restraint session was 30 min, after which the mouse was returned to its home cage. The device was cleaned using 70% ethanol between mice. To reduce variation due to handling, the same researcher performed all of the restraint stress. To account for the additional handling and transport stress that accompanied movement to and from the restraint room, mice from the control group were transported an equal distance but without undergoing the restraint stress.

### Forced Swim Stress

The forced swim stress (FSS) consisted of a plastic cylinder (69 cm diameter) filled with room-temperature water at a depth of 33 cm. To perform the FSS, a mouse cage was moved into the stress designated room in which the FSS was stored. The mouse was allowed to acclimate in its cage for 30 min in the room in which the FSS was conducted. All mice were placed into the same location (center) of the FSS cylinder and at the same directional orientation. The mouse was gently placed into the water and allowed to swim or float for 15 min under continuous supervision. Following the FSS, the mouse was placed onto a clean absorbent towel for 15 min in a clean cage and visually confirmed to dry completely before being returned to its home cage. To account for the additional handling and transport stress that accompanied movement to and from the restraint room, mice from the control group were transported an equal distance in a fresh cage but without undergoing the FSS.

### Elevated Plus Maze

The elevated plus maze (EPM) platform was 50 cm above the floor and consisted of four arms (two walled and two un-walled arms) of equal length (35 cm). The central platform was 25 cm^2^. To perform the EPM, a mouse was gently placed onto the center platform facing a walled arm. Each mouse was oriented at the beginning of the EPM toward the same walled arm. Each mouse was tested in the EPM for a single period of 10 min. The EPM was cleaned using 70% ethanol between each mouse. To reduce an effect of handling variability, the same researcher performed all behavioral experiments. A camera (HD ProWebcam C920) was mounted directly above the EPM to record the testing field. Each testing session was recorded using the AnyMaze™ software (v6.10, Stoelting, MA). The equivalent of four paws (85% of the mouse’s body) was used as the criterion for an entry into an arm of the EPM. All recorded behavior was manually scored by a blinded researcher.

### Barnes Maze

The Barnes maze was conducted as previously described (Paul et al., 2009). In brief, the maze (Maze Engineers; Skokie, IL) consisted of 20 equally spaced holes each 5 cm in diameter with a single black nesting box. The maze was 92 cm in diameter and 95 cm above the floor. The test, and all training sessions, were conducted under standard room lighting. Before the start of the training period, mice were habituated for 30 min in the room that housed the Barnes maze. Mice were introduced to the Barnes Maze apparatus once before beginning the training process and gently guided by the handler to the escape hole. Training consisted of 4 days where each day was composed of four 3 min trials, with 15 min of rest separating each trial. The maze was cleaned with 70% ethanol between each mouse visit. To assess a short-term and long-term effect of stress on memory, mice were tested first on the day immediately after training completion and then again 7 days post-training.

### Tissue Collection

Mice were gently removed from their home cage and placed into a clean unused cage for a period of 30 min which was sufficient time to produce fecal pellets. All fecal pellets were aseptically collected and stored at −80°C until genomic DNA extraction. Immediately following sacrifice all intestinal tissues were carefully opened longitudinally with a ball-tipped scissor and intestinal content gently removed. Beginning at the proximal end of each intestinal section, sections of full thickness duodenum, jejunum, ileum, and proximal and distal colon were collected as this consistently gave the required 100–200 mg for neurochemical analyses. Cecal content was aseptically collected into sterile microfuge tubes, snap frozen on dry ice and stored at −80°C until DNA extraction. Cecal tissue was collected for neurochemical analysis. As previously described (Bauer et al., 2021) all intestinal tissue samples were immediately acidified in 0.2N perchloric acid and stored at −80°C until Ultra High Performance Liquid Chromatography with Electrochemical Detection (UHPLC-ECD) analysis.

### Ultra High Performance Liquid Chromatography With Electrochemical Detection

Tissues were thawed and then homogenized twice for 30 s at 5 m/s in a Bead Ruptor (Catalog #: 19-040E, Omni International) before centrifugation at 3,000 × g at 4 °C for 15 min. Supernatant was purified through 2–3 kDA molecular weight cut-off spin filters (Catalog #: 89132-006, VWR Life Science) after which flow-through was collected and stored at − 80 °C until analysis by UHPLC-ECD as previously described (Bauer et al., 2021). The UHPLC-ECD setup included a Dionex Ultimate 3000 autosampler, a Dionex Ultimate 3000 pump, and a Dionex Ultimate 3000 RS electrochemical detector (Thermo Fisher Scientific, Sunnyvale, CA). Mobile phase consisted of buffered 10% acetonitrile (Catalog #: NC9777698, Thermo Fisher Scientific), and the flow rate was 0.6 mL/min on a 150-mm (length), 3-mm (internal diameter), and 3-μm (particle size) Hypersil BDS C18 column (Catalog #: 28103-153030, Thermo Fisher Scientific). All samples were held under 4 °C on the autosampler prior to injection, and electrochemical detection was performed using a 6041RS glassy carbon electrode set at 400 mV. The Chromeleon software package (version 7.2, Thermo Fisher Scientific) was used to analyze data, and neurochemical identification was confirmed using relative retention times of corresponding analytical standards from Millipore-Sigma (for serotonin, Catalog #: 61-47-2; for 5-hydroxyindoleacetic acid (5-HIAA), Catalog #: 54-16-0).

### Genomic DNA Isolation

Total DNA was isolated using the DNeasy PowerSoil Kit (Qiagen, Catalog #12988-10) with the following modifications: a total of 250 mg cecal content or fecal pellets was used as input, and samples were vortexed for 20 min using bead-containing tubes. Extracted DNAs were assessed for quality using a NanoDrop 2000 spectrophotometer 260–280 nm ratios. Concentrations were determined using a Qubit fluorometer with the double-stranded DNA broad range kit (Thermo Fisher Scientific), adjusted to 50 ng/μl in nuclease-free water, and shipped on dry ice to Argonne National Laboratory in Lemont, Illinois. All cecal and fecal samples were used for sequencing. DNAs were used for library preparation using the MiSeq and HiSeq2500 kit (Illumina) following all manufacturer’s instructions with 151 × 151 paired-end sequencing.

PCR amplicon libraries targeting the 16S rRNA gene present in extracted DNA were produced using a barcoded primer set adapted for Illumina MiSeq (Caporaso et al., 2012). DNA sequence data was generated using Illumina MiSeq paired-end sequencing at the Environmental Sample Preparation and Sequencing Facility (ESPSF) at Argonne National Laboratory (Lemont, IL, United States). Specifically, the V4 region of the 16S rRNA gene (515F–806R) was PCR amplified with region-specific primers that included sequencer adaptor sequences used in the Illumina MiSeq flowcell (Caporaso et al., 2012). The forward amplification primer also contained a twelve base barcode sequence that supports pooling of up to 2,167 different samples in each lane (Caporaso et al., 2011, 2012). Each 25 μL PCR reaction contained 9.5 μL of MO BIO PCR Water (Certified DNA-Free), 12.5 μL of QuantaBio’s AccuStart II PCR ToughMix (2x concentration, 1x final), 1 μL Golay barcode tagged forward primer (5 μM concentration, 200 pM final), 1 μL reverse primer (5 μM concentration, 200 pM final), and 1 μL of template DNA. The conditions for PCR were as follows: 94°C for 3 min to denature the DNA, with 35 cycles at 94 °C for 45 s, 50 °C for 60 s, and 72 °C for 90 s; with a final extension of 10 min at 72 °C to ensure complete amplification. Amplicons were then quantified using PicoGreen (Invitrogen) and a plate reader (Infinite 200 PRO, Tecan). Once quantified, volumes of each of the products were pooled into a single tube so that each amplicon was represented in equimolar amounts. This pool was then cleaned up using AMPure XP Beads (Beckman Coulter), and then quantified using a fluorometer (Qubit, Invitrogen). After quantification, the molarity of the pool was determined and diluted down to 2 nM, denatured, and then diluted to a final concentration of 6.75 pM with a 10% PhiX spike for sequencing on the Illumina MiSeq. Amplicons were sequenced on a 151bp MiSeq run using customized sequencing primers and procedures (Caporaso et al., 2012).

### 16S rRNA Gene Amplicon Sequence Analysis

Sequence analysis was performed using Qiime2 (release 2-2019.10) following the steps described in the Moving Pictures and Atacama Soil tutorials (Bolyen et al., 2019). Barcode sequences and primers were removed upon importing the raw reads, and forward and reverse reads were truncated to 145 and 140 bases, then 5 bases were trimmed from the 5′ end from both forward and reverse reads to remove poor quality segments. Chimeric sequences were detected and removed using the “consensus” chimera-method option within the DADA2 denoising step. Amplicon Sequencing Variants (ASVs) were generated with the internal DADA2 tool, and the SILVA SSU NR reference database (V132) was used to assign taxonomy to each ASV. The ASV counts table and taxonomy assignments were then imported into R and analyzed using Phyloseq (v1.34.0; McMurdie and Holmes, 2013) and Vegan (v2.5-5; Oksanen et al., 2020). One cecal and two fecal samples were removed from analysis due to insufficient read depth.

### Statistics

UHPLC and behavioral data were analyzed using GraphPad Prism (v. 9.1.0; La Jolla, CA, United States). Two-way ANOVA with Tukey’s honest significant difference (HSD) *post-hoc* test was used to correct for multiple comparisons. Normality was assessed using the Shapiro-Wilk test. Data are presented as mean ± SEM. Results were considered statistically significant at *p* < 0.05.

Bacterial community data comparing the effects of diet, sex and stress was analyzed according to the following statistical model:


(1) $$Y_{i\,j\,k\,l}=\mu +d_{i}+x_{j}+t_{k}+d_{i}\,x_{j}+d_{i}\,t_{k}+x_{j}\,t_{k}+d_{i}\,x_{j}\,t_{k}+e_{i\,j\,k\,l}$$

Where *Y*_*ijkl*_ is the observed value for kth experimental unit within the *_*i*_*th level of diet (chow diet vs. beef-chow mix diet) at the *_*j*_*th sex (Male vs. Female) and *_*k*_*th level of stress (yes vs. no); μ is the general mean; *d*_*i*_ is the fixed effect of the *_*i*_*th diet (*i* = chow diet vs. beef-chow mix diet); *x_j_* is the fixed effect of the *_*j*_*th sex (*j* = Male vs. Female); *t_k_* is the fixed effect of the *_*k*_*th level of stress (*k* = yes vs. no); *d*_*i*_*x*_*j*_ is the subsequent interaction of diet and sex; *d*_*i*_*t*_*k*_ is the subsequent interaction of diet and stress; *x*_*j*_*t*_*k*_ is the subsequent interaction of sex and stress; *d*_*i*_ *x_j_t_k_* is the subsequent interaction of diet, sex, and stress; and *e*_*ijkl*_ is the associated variance as described by the model for *Y*_*ijkl*_ (l = 1 through 77).

Estimates of Chao species richness, Shannon diversity, and Simpson evenness were taken to compare community structures between experimental groups. The means of the experimental group alpha diversity measures were analyzed using the PROC MIXED procedure according to the described model (Eq. 1). Additionally, Bray-Curtis dissimilarity between experimental groups were analyzed using the Adonis command (PERMANOVA) and BetaDisp command (BetaDispersr) provided within the Vegan (v2.5-5; Oksanen et al., 2020) package according to the same model. All pairwise contrasts between levels within the independent variables were made using pairwiseAdonis (v0.4; Martinez, 2020) Overall variation in bacterial communities was visualized using Principal Coordinates analysis (PCoA). Canonical analysis of principal coordinates (CAP; Anderson and Willis, 2003) was used to visualize the variation based on the model proposed above. This information was generated with the Phyloseq (v1.34.0; McMurdie and Holmes, 2013) and Vegan (Oksanen et al., 2020) packages. All plotting was completed using ggplot2, v2_3.1.1 graphing package in R 4.1.0.

Additionally, the abundance of the 100 most abundant ASVs among samples was analyzed using a negative binomial distribution in GLIMMIX procedure of SAS (Version 9.4, SAS Inst., Cary, NC) according to the model described above (Eq. 1). All count data were offset by the total library count for a given sample. Corresponding *p*-values were corrected for false discovery rates using the MULTITEST procedure of SAS. Least square means were separated using Fisher’s Least Significant Difference test, and treatment differences were considered significant if *p* (or *q*) values were < 0.05. For the top 100 ASVs with a treatment *q* value of < 0.05, the log2-fold change was calculated comparing male or female animal groups experiencing stress or not while fed chow diet or beef-chow mix diet. Throughout the manuscript, the authors will refer to bacterial community structure when describing richness, evenness and diversity measurements and bacterial community composition when referring to shifts in ASV abundance and Bray-Curtis dissimilarity.

## Results

### Diet Mediates Gut Serotonergic Responses to Stress in a Sex-Dependent Manner

Distal colonic serotonin concentrations were not significantly affected by stress (main effect of stress *p* = 0.072) in male mice regardless of diet type (Table 1). Conversely, female mice (Table 2) that received a beef-supplemented diet but not those that consumed the chow only diet exhibited an increase (main effect of diet *p* = 0.223; main effect of stress *p* = 0.001) in distal colonic serotonin. Stress caused an increase in proximal colonic serotonin in male and female mice that were provided the beef diet, but not in either sex that received the chow diet (for males, main effects of stress (*p* = 0.017) and diet (*p* = 0.002); for females, main effects of stress (*p* = 0.003) and diet (*p* = 0.037). Stress elicited an increase in cecal serotonin concentrations in female but not male mice, and only in those female mice that received the beef diet (for males, main effects of stress (*p* = 0.696) and diet (*p* = 0.888); for females, main effects of stress (*p* = 0.003) and diet (*p* = 0.037). Likewise, female small intestinal serotonin, including the ileal and duodenal regions, was elevated in stressed mice that receive the beef-diet but not in the stressed chow group.

**TABLE 1:** Serotonin and 5-HIAA concentrations in the male intestinal tract exhibit diet-dependent responsive to chronic stress.

		Duodenum	Jejunum	Ileum	Cecum	Proximal colon	Distal colon
Group	Analyte						
Chow	Serotonin	1.796 ± 0.282	1.350 ± 0.206^#^	0.792 ± 0.186^#^	1.386 ± 0.334	1.600 ± 0.385	5.886 ± 1.215^#^
	5-HIAA	2.699 ± 0.262	1.771 ± 0.281	2.506 ± 0.340	9.834 ± 1.050	17.360 ± 3.084	7.858 ± 1.135*
Chow + Stress	Serotonin	2.537 ± 0.357	1.639 ± 0.277	0.820 ± 0.099	0.926 ± 0.170	1.717 ± 0.283^#^	7.531 ± 1.287
	5-HIAA	3.105 ± 0.287	2.232 ± 0.305	2.465 ± 0.244	11.715 ± 1.678^#^	19.191 ± 1.732	14.238 ± 1.286^#^
Chow-Beef	Serotonin	0.848 ± 0.120	0.353 ± 0.071	0.184 ± 0.092	0.782 ± 0.203	2.169 ± 0.656*	1.851 ± 0.387
	5-HIAA	2.096 ± 0.441	2.270 ± 0.188	2.294 ± 0.253	7.633 ± 0.736	13.763 ± 0.778	6.711 ± 0.860
Chow-Beef + Stress	Serotonin	1.554 ± 0.218	0.951 ± 0.197	0.616 ± 0.133	1.454 ± 0.329	5.100 ± 0.940	4.099 ± 0.936
	5-HIAA	2.083 ± 0.236	2.468 ± 0.190	2.927 ± 0.649	6.807 ± 0.789	12.577 ± 1.942	7.235 ± 1.070

*Denotes significant difference (p < 0.05) within same tissue region of same diet/stress groups (e.g. chow vs. chow + stress or chow-beef vs. chow-beef + stress).

*^#^Denotes significant difference (p < 0.05) within same tissue region within stress or control groups of different diets (e.g., chow vs. chow-beef or chow + stress v chow-beef + stress). Values are μg of serotonin or 5-HIAA per g of tissue. All values are expressed as mean ± SEM (n = 9–10 mice/group). Data was analyzed using two-way ANOVA followed by Tukey’s post-hoc test as described in section “Materials and Methods.” 5-HIAA: 5-hydroxyindoleacetic acid. Stress encompassed a chronic alternating forced swim and restraint stress paradigm as described in section “Materials and Methods.”*

**TABLE 2:** Serotonin and 5-HIAA concentrations in the female intestinal tract exhibit diet-dependent responsive to chronic stress.

		Duodenum	Jejunum	Ileum	Cecum	Proximal colon	Distal colon
Group	Analyte						
Chow	Serotonin	1.091 ± 0.154^#^	0.796 ± 0.103	0.505 ± 0.066^#^	0.759 ± 0.215	2.608 ± 0.531	1.351 ± 0.264
	5-HIAA	0.695 ± 0.086	0.924 ± 0.158	1.065 ± 0.173	3.872 ± 0.431	2.287 ± 0.452	1.815 ± 0.223
Chow + Stress	Serotonin	1.359 ± 0.090	0.929 ± 0.172	0.606 ± 0.099	1.199 ± 0.178	3.526 ± 0.322	2.490 ± 0.443
	5-HIAA	0.614 ± 0.038	0.990 ± 0.127	1.521 ± 0.200	4.524 ± 0.454	3.240 ± 0.481	1.955 ± 0.088
Chow-Beef	Serotonin	0.490 ± 0.128*	0.341 ± 0.103	0.120 ± 0.083*	0.276 ± 0.161*	0.829 ± 0.507*	0.308 ± 0.214*
	5-HIAA	0.696 ± 0.107	1.006 ± 0.201	1.186 ± 0.083	3.334 ± 0.473	2.352 ± 0.351*	0.876 ± 0.126
Chow-Beef + Stress	Serotonin	1.046 ± 0.080	0.810 ± 0.140	0.481 ± 0.060	1.246 ± 0.240	3.093 ± 0.636	2.401 ± 0.700
	5-HIAA	0.687 ± 0.082	0.838 ± 0.070	1.266 ± 0.126	3.574 ± 0.674	4.211 ± 0.577	2.546 ± 0.869

*Denotes significant difference (p < 0.05) within same tissue region of same diet/stress groups (e.g., chow vs. chow + stress or chow-beef vs. chow-beef + stress).

^#^Denotes significant difference (p < 0.05) within same tissue region within stress or control groups of different diets (e.g., chow vs. chow-beef or chow + stress v chow-beef + stress). Values are μg of serotonin or 5-HIAA per g of tissue. All values are expressed as mean * SEM (n = 9–10 mice/group). Data was analyzed using two-way ANOVA followed by Tukey’s post-hoc test as described in section “Materials and Methods.” 5-HIAA: 5-hydroxyindoleacetic acid. Stress encompassed a chronic alternating forced swim and restraint stress paradigm as described in section “Materials and Methods.”

Stress elicited an increase in male but not female distal colonic 5-HIAA only in the chow-group (for males, main effects of stress (*p* = 0.003) and diet (*p* = 0.0009); for females, main effects of stress (*p* = 0.059) and diet (*p* = 0.709). In the proximal colon, stress caused an increase in 5-HIAA in female mice (main effect of stress, *p* = 0.005) that received the beef diet, but not in male mice regardless of diet (main effect of stress, *p* = 0.880). Cecal 5-HIAA concentrations diverged between male mice which received different diets and also underwent stress (main effects of stress (*p* = 0.653) and diet (*p* = 0.004)). Small intestinal 5-HIAA concentrations were not significantly affected by stress or diet regardless of sex.

### Cecal Bacterial Communities

#### An Overview of the Cecal Bacterial Sequencing Dataset

When considering the entire cecal dataset, 996 ASVs were generated (one sample was removed due to low sequencing depth) after quality control and removal of ASVs representing less than 10 sequences. The average sequencing depth per sample was 21,577 sequences with a standard deviation of 6,540 sequences. Overall, 8 phyla were discovered within the cecal bacterial community. Of which, *Firmicutes*, *Bacteroidetes*, and *Proteobacteria* were the most abundant, representing 70.9, 21.7, and 4% of all reads, respectively (Supplementary Table 1 and Supplementary Figure 1). The abundance and classifications of the 50 most abundant ASVs can be found in Supplementary Table 2.

#### Host Sex Influences Cecal Bacterial Community Structure

**FIGURE 1:**
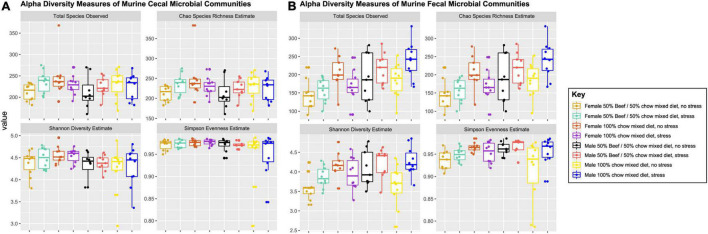
Measures of alpha-diversity indices of cecal **(A)** and fecal **(B)** murine bacterial communities visualized for every combination of the fixed effects diet, sex and stress. Stress encompassed a chronic alternating forced swim and restraint stress paradigm as described in section “Materials and Methods.” Error bars 1.5X the 75th (upper) and 25th (lower) percentile. Significance of main effects and interactions were tested using the models described in the statistics section (Eqs 1 and 2) and are listed in Supplementary Table 3.

After correction for multiple testing using Tukey’s HSD, the fixed effects of diet and stress did not affect cecal bacterial community structure (Chao species richness, Simpson evenness and Shannon diversity). However, the fixed effect of sex had a significant effect on Simpson evenness (*p* = 0.03) and Shannon diversity (*p* = 0.015) in cecal bacterial communities, demonstrating female mice had higher Simpson evenness and Shannon diversity estimates than was seen in males (Supplementary Table 3 and Figure 1).

#### Interplay of Host Sex, Diet, and Stress Impacts Cecal Bacterial Community Composition

**FIGURE 2:**
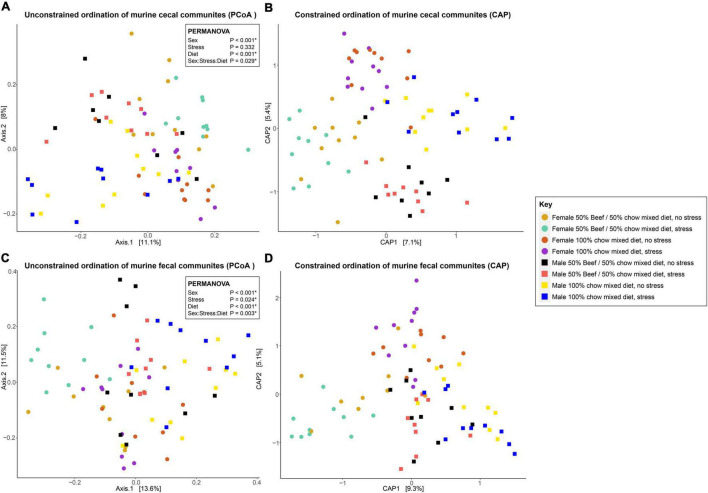
Unconstrained (principal coordinates analysis, PCoA) ordinations of cecal **(A)** and fecal **(C)** bacterial communities, and constrained (canonical analysis of principal coordinates, CAP) ordinations comparing murine cecal **(B)** and fecal **(D)** bacterial communities visualized by the fixed effects of diet, sex and stress. Stress encompassed a chronic alternating forced swim and restraint stress paradigm as described in section “Materials and Methods.” Distances between samples denote Bray-Curtis dissimilarity measures. CAP analysis was constrained by Eq. 1.

Although the fixed effects of diet and sex did not significantly affect community structure, they did affect community composition. A three-way interaction between diet, sex and stress had a significant effect on the Bray-Curtis dissimilarity distribution of groupings detected by PERMANOVA (*p* = 0.029) and visualized by PCoA (Supplementary Table 4 and Figure 2). Pairwise comparisons, including the default Bonferroni’s correction for multiple testing, were then made to determine which groupings of these variables significantly differed from one another. Diet changes in female mice significantly (*q* > 0.05) affected the cecal bacterial communities regardless of stress, but in female groups fed the same diet, stress did not drive distinguishable changes in cecal bacterial community composition (Supplementary Table 5 and Figure 2). When stressed, female cecal bacterial communities from animals fed chow only diets are indistinguishable (*q* > 0.05) from non-stressed females fed the beef supplementation diet based on these pairwise PERMANOVA analysis (Supplementary Table 5 and Figure 2). Nearly all comparisons between male and female cecal bacterial community composition were significantly different, barring a few specific combinations of fixed effects. Of note, when male and female mice fed the same diet and were not stressed, bacterial community composition was indistinguishable. Only when stress was applied to one or both groups were they distinguishable, except for non-stressed female mice and stressed male mice fed the beef diet. All individual comparisons of cecal bacterial community composition across the levels of fixed effects sex, diet and stress are included in Supplementary Table 5.

The abundance of the 100 most abundant cecal ASVs were compared using the model described below (Eq. 1), and a false discovery rate (FDR) correction for multiple testing was applied *post-hoc*. ASVs 16 (*Desulfovibrio*) and 52 (*Clostridium* sp. *Culture-41*) were more abundant in females whereas 30 (*Bifidobacterium*) and 46 (*Ruminococcaceae UCG-014*) were more abundant in males (Supplementary Table 6). ASVs 6 (*Lactobacillus murinus*) and 58 (*Lactobacillus murinus*) were more abundant in animals fed the beef supplemented diet compared to mice fed the standard chow diet (Supplementary Table 7).

### Fecal Bacterial Communities

#### An Overview of the Fecal Bacterial Sequencing Dataset

When considering the entire fecal dataset, 949 ASVs were generated (two samples were removed due to low sequencing depth) after quality control and removal of ASVs representing less than 10 sequences. The average sequencing depth per sample was 22,770 sequences with a standard deviation of 9,177 sequences. Similar to the cecal community, 8 phyla were discovered within the fecal bacterial community. Of which, *Firmicutes*, *Bacteroidetes*, and *Actinobacteria* were the most abundant, representing 55, 38.2, and 2.6% of all reads, respectively (Supplementary Table 8 and Supplementary Figure 1). The abundance and classifications of the 50 most abundant fecal ASVs can be found in Supplementary Table 9.

#### Interplay of Host Sex, Diet, and Stress Impacts Fecal Bacterial Community Structure and Composition

Chao species richness was significantly affected by a sex*stress interaction (*p* = 0.001), and both Simpson evenness (*p* = 0.03) and Shannon diversity (*p* = 0.008) measures were significantly impacted by a three-way (diet, sex, and stress) interaction effect (Supplementary Table 10 and Figure 1). Pairwise comparisons with a Tukey’s HSD correction for multiple testing were conducted to determine which group significantly differed from one another. Females, either stressed or non-stressed had lower Chao species richness than male mice (*q* < 0.01) that were stressed (Supplementary Table 11). Additionally, non-stressed females fed the chow-only diet had greater Simpson evenness (*q* = 0.016) and non-stressed females fed the beef diet had lower Shannon diversity than stressed males on either diet (*q* < 0.035).

Fecal bacterial communities were significantly affected by the fixed effects of stress, diet, and sex with an overarching interactive effect (*p* = 0.003) between all three variables when using PERMANOVA (Supplementary Table 12 and Figure 2). When pairwise comparisons were made between these communities, diet only associated with changes when stress was applied in either group (or both at the same time). No changes were identified for stress within groups fed the same diet either, demonstrating the interactions between variables (Supplementary Table 13). In males, differences in fecal bacterial communities were only observed between diets when both groups were stressed (*q* = 0.028). All other community combinations were indistinguishable (Supplementary Table 13). As with the cecal data set, all individual comparisons of fecal bacterial community composition across the levels of fixed effects sex, diet and stress are included in Supplementary Table 13.

The abundance of the 100 most abundant fecal ASVs was compared using the model described previously (Eq. 1), and an FDR correction for multiple testing was applied *post-hoc*. ASVs 1 (*Lactobacillus*), 2 (*Muribaculaceae*), 5 (*Lactobacillus*), 6 (*Lactobacillus murinus*), 8 (*Muribaculaceae*), 24 (*Alistipes*), 37 (*Muribaculaceae*), 45 (*Lachnospiraceae NK4A136 group*), 52 (*Clostridium* sp. *Culture-41*), 58 (*Lactobacillus murinus*), 64 (*Ruminiclostridium 9*), and 193 (*Enterorhabdus*) were more abundant in female mice and ASV 91 (*Muribaculum*) was more abundant in male mice (Supplementary Table 14). ASVs 6 (*Lactobacillus murinus*), 27 (*Bacteroides*), 58 (*Lactobacillus murinus*), 83 (*Parasutterella*), and 101 (*Staphylococcus*) were more abundant in animals fed beef supplemented chow, whereas ASVs 12 (*Lactobacillus*) and 114 (*Muribaculaceae*) were more abundant in mice fed the standard chow diet (Supplementary Table 15). ASVs 30 (*Bifidobacterium*) and 146 (*Bifidobacterium*) were affected by a sex*diet interaction, with ASV 30 being more abundant in female mice fed standard chow compared to female mice fed beef supplemented chow and male mice fed standard chow and being more abundant in male mice fed beef supplemented chow diets over females fed beef supplemented diets. Additionally, ASV 146 was more abundant in male mice compared to female mice when both were fed the standard chow diet (Supplementary Table 16).

#### Diet Modulates the Impact of Stress on Anxiety-Like Behavior and Memory

**FIGURE 3:**
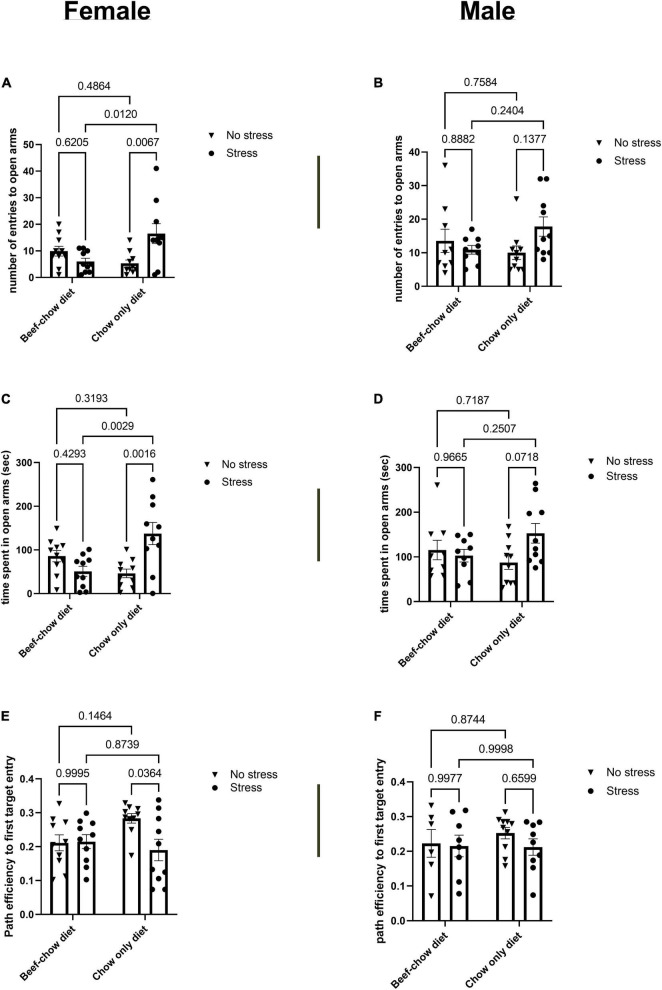
Behavioral outcomes following chronic alternating stressors are sex- and diet- dependent. Number of entries to open arms in the elevated plus maze for female **(A)** and male mice **(B)**. Time spent in open arms of elevated plus maze for female **(C)** and male **(D)** mice. Long term memory path efficiency to first target entry for female **(E)** and male **(F)** mice. Individual values displayed on each bar graph comparison are *p*-values. Data was analyzed using two-way ANOVA followed by Tukey’s *post-hoc* test as described in section “Materials and Methods.” Stress encompassed a chronic alternating forced swim and restraint stress paradigm as described in section “Materials and Methods.”

To determine changes in anxiety-like behavior, mice were tested on the elevated plus maze (EPM) (Figure 3). Female but not male mice that underwent stress and received the chow diet displayed increased number of entries to open arms and time spent in open arms compared to control chow group (*p* = 0.001 for time spent in open arms; *p* = 0.006 for entries to open arms). The latency to first visit of the open arms did not differ between any group regardless of sex. Parameters measured in the EPM closed arms were not affected by diet, sex, or stress. To assess changes in short- and long-term memory, mice were evaluated to the Barnes maze (Figure 3). Differences in short-term memory were not found due to diet, sex, or stress. Path efficiency to first target entry was reduced (*p* = 0.036) in stressed female mice that received the chow diet compared to non-stress controls. Other parameters used to evaluate long-term memory were not significantly affected.

## Discussion

The neurobiology of stress responsivity is, in part, affected by the gut microbiome as well as determined by host diet and inherent sex-related differences in neuroendocrine circuitry. Adding to the complexity of the stress response is that neuroendocrine axes serve as causal routes of bi-directional communication between host and microbiome, thereby enabling host sex differences that characterize neuroendocrine signaling pathways to affect microbiome community dynamics. Yet, little is known regarding the role of host sex in determining microbiome responsivity to chronic stress and eliciting changes in intestinal neurochemical concentrations that have demonstrated roles in affecting host behavior via the microbiota-gut-brain axis. The results presented herein demonstrate that host sex and diet play determinative roles in driving both host and microbiome responses to chronic stress, including region-dependent intestinal serotonergic responses and distinct compositional changes in the cecal and fecal bacterial communities. In addition, behavioral responses to stress that diverged according to host sex were observed to be altered by diet. Together these results indicate that the impact of chronic stress on the microbiota-gut-brain axis is dependent on host sex and may be modifiable by diet, ultimately underscoring the need to include both sexes in microbiome-related stress research.

Peripheral serotonergic signaling plays myriad roles in intestinal function (Jones et al., 2020) and is increasingly understood to be of importance in chronic stress-related gut disorders that involve the microbiome and are tailorable by diet (O’Mahony et al., 2015) such as irritable bowel syndrome (Gros et al., 2021; Mishima and Ishihara, 2021), that disproportionately affect women compared to men (Spiegel, 2009). In the present study we identified that sex-dependent intestinal serotonergic responses occur following chronic stress. Surprisingly, few studies have investigated the impact of psychosocial stress on intestinal serotonin concentrations. Previously it was demonstrated that the sensitivity of the enteric serotonergic system to a single acute stressor was dependent on the presence of the microbiome and that proximal colonic serotonin concentrations increased following stress only in chow-fed male but not female C57/BL6 mice (Lyte et al., 2020). Our findings confirm that this effect of stress on colonic serotonin concentrations occurs also following chronic stress, but that mouse strain plays a role in determining sex-specific susceptibility of the regional colonic serotonergic responses to stress; we observed this effect in the proximal colon of both male and female CF-1 mice, however, in the distal colon only in female mice. Indeed, previous evidence (Browne et al., 2011) demonstrated mouse strain may determine distinct central serotonergic responses to stress.

It is important to highlight that the colonic serotonergic stress responses were only observed in mice that received a beef-supplemented chow diet, especially as the chow diet employed in the present study contained no animal protein. As serotonin in the gut can mediate inflammation (Gershon, 2012), and stress episodes have been noted to precede IBS symptom flare-ups (Qin et al., 2014), our findings suggest stress-based changes in enteric serotonin are adjustable through diet. Indeed, the ratio of meat and plant-based food intake was identified to play a key role in the enrichment of bacterial taxa that associate with IBS symptom severity in patients (Tap et al., 2021). Conversely, a recent study that only utilized chow-fed C57/BL6 male mice, observed chronic restraint stress to reduce colonic serotonin concentrations (Deng et al., 2021).

Beyond the colon, we examined serotonergic changes in other regions of the gut. Similar to our findings in the colon, cecal and ileal serotonin increased following stress in the chow-beef mix fed female but not male mice. While the use of germ-free mice has been used for decades to demonstrate the impact of the microbiota in determining, for example rodent ileal, cecal, and colonic serotonin concentrations (Hata et al., 2017; Lyte et al., 2020), only a single study has examined the role of the microbiota in determining enteric serotonergic stress responsivity. This may suggest that the selective targeting of specific members of the microbiota, through the use of antibiotics or other means, may blunt enteric serotonin responses. Bacterial taxa reported to modulate tryptophan metabolism, including *Clostridium* spp. (Deng et al., 2021), were found in greater abundance in the cecal microbiota of female mice. The findings of the present study warrant further examination of the role of the microbiome in determining sex-specific serotonin responses to chronic stress that are dependent on diet.

Like regional differences in enteric serotonin, the microbiome exhibits spatial differences in taxa membership depending on location within the gut (Tropini et al., 2017). It is well-recognized that both the fecal and cecal microbial communities are responsive to host stress, but little investigation has been performed into the interaction of sex and diet in mediating stress-induced regional changes in microbiota composition. We identified that the fecal bacterial communities, which were indistinguishable between same-sex control group mice that received different diets, became compositionally distinct following stress. This may indicate that different host diets distinctly impact microbiome functionality, imparting changes in microbial susceptibility or resilience to stress that are not identifiable using compositional approaches that rely on a single gene (e.g., 16S) but instead require investigation of the complete bacterial genome (e.g., whole genome shotgun sequencing). Our findings suggest that host diet may prime the responsivity of the fecal microbiota to stress. Specific taxa known to modulate host gut serotonin concentrations, including *Bacteroides* (Yano et al., 2015), were found to be more abundant in mice that received the beef diet. As various studies have demonstrated that the composition of the microbiota can be modulated by serotonin (Kwon et al., 2019) and specific microbial taxa can uptake serotonin (Lyte and Brown, 2018), and we observed colonic serotonin concentrations to change only in beef-supplemented groups that underwent stress, future studies should examine whether stress-induced changes in serotonergic transmission in the gut contribute in driving the divergence of the microbiome following stress. Conversely, in the cecum, diet was found to be the dominant factor in driving compositional changes in the female but not male bacterial communities. While sex-specific effects of diet on the gut microbiota have been previously described (Bolnick et al., 2014), relatively few studies have examined the effect of dietary inclusion of beef on the female microbiota (Albracht-Schulte et al., 2021).

Intestinal concentrations of serotonin have been known for decades to stimulate vagal afferents (Zhu et al., 2001) that contribute to the microbiota-gut-brain axis (Bonaz et al., 2018). As stress has been shown to mediate anxiety-like behavior and alterations in working memory that associate with changes in microbial taxa, we sought to identify whether alterations in enteric serotonin concentrations coincided with behavioral outcomes. Of note, in the elevated-plus maze female mice that had received the chow-only diet and had undergone stress spent more time in the open arms, a measure indicative of anxiolytic behavior (Kraeuter et al., 2019), compared to female mice that were stressed and had received the beef supplemented chow diet. Whereas colonic serotonin increased following stress in the beef-supplemented chow group of female mice, it did not change compared to controls in the chow-only female group, suggesting diet dependent serotonin changes in the gut coincide with anxiety-like behavior in female mice. While this was an unexpected finding, behavioral tests that rely on rodent aversion to open, brightly lit spaces such as the elevated plus or T-maze have been widely used to validate the role of serotonin in anxiety (Zangrossi, and Graeff, 2014). Sex-dependent differences in rodent performance as well as display of anxiogenic behavior as a result of sexually divergent sensitivity to chronic stress paradigms have previously been described (Mitra et al., 2005; Knight et al., 2021). Furthermore, whereas the chow-only group of female mice displayed a reduced pathfinding efficiency, indicative of working memory, compared to respective control, female mice that were stressed but received a beef-supplemented diet did not differ in this aspect from their control group which did not undergo stress. Indeed, tryptophan supplementation, an amino acid found in beef, has been shown to affect memory during stress (Li et al., 2009; Hulsken et al., 2013), and the modulation of serotonin signaling in the gut has been demonstrated to both affect rodent behavior and mediate anxiety-like behavior. Early elevation of central serotonin that occurs during initial selective serotonin reuptake inhibitor (SSRI) treatment have been demonstrated to acutely exacerbate anxiety (Frick et al., 2015; Marcinkiewcz et al., 2016). Separately, administration of fluoxetine, an SSRI, was shown to increase local serotonin production in the rodent colon (Kannen et al., 2011), as well as mediate anxiety-like behavior (Lyte et al., 2019). As a recent investigation in rodents demonstrated that the SSRI class of psychiatric drugs, in part, act from the gut via the vagus nerve to affect behavior via the microbiota-gut-brain axis (McVey Neufeld et al., 2019), it is plausible that acute increases in enteric serotonin may affect host anxiety-like behavior. Moreover, as fluoxetine has been demonstrated to affect the microbiome, and that the microbiome affects host serotonin synthesis, it would be pertinent to identify the role of microbial contributions to serotonin-mediated alterations in host behavior that follow stress or dietary changes.

As the microbiota-gut-brain axis is bi-directional, host characteristics such as age, genetics, and other factors collectively shape the microbiome. Less is understood, however, whether host sex may influence the impact of stress, diet, or other important environmental effects on the microbiota. The results presented herein demonstrate that host sex and diet interact to modulate intestinal serotonergic and bacterial community responses to stress. As serotonergic concentrations in the gut mediate, in part, bi-directional communication between microbiota and host that may ultimately influence host behavior, chronic stress was here identified to distinctly affect male and female behavior. Together, our findings underscore the need to utilize both sexes in the study of the microbiota-gut-brain axis.

## Data Availability Statement

The datasets presented in this study can be found in online repositories. The names of the repository/repositories and accession number(s) can be found below: The 16S rRNA gene sequences have been submitted to the NCBI Sequence Read Archive SRA and are available under the BioProject ID PRJNA753915.

## Ethics Statement

The animal study was reviewed and approved by the Iowa State University Institutional Animal Care and Use Committee.

## Author Contributions

ML conceived, designed the study, and edited the manuscript. KD performed neurochemical and behavioral studies, curated, analyzed the data, and edited the manuscript. LK performed all microbiome data analyses, wrote, and edited the manuscript. JL contributed to the design of the study, performed all neurochemical biogeography analyses, wrote, and edited the manuscript. All authors contributed to the article and approved the submitted version.

## Conflict of Interest

The authors declare that the research was conducted in the absence of any commercial or financial relationships that could be construed as a potential conflict of interest.

## Publisher’s Note

All claims expressed in this article are solely those of the authors and do not necessarily represent those of their affiliated organizations, or those of the publisher, the editors and the reviewers. Any product that may be evaluated in this article, or claim that may be made by its manufacturer, is not guaranteed or endorsed by the publisher.

## Supplementary Material

Supplementary Figure 1 Relative abundance of 5 most abundant phylum for cecal **(A)** and fecal **(B)** murine bacterial communities, visualized for every combination of the fixed effects diet, sex and stress. Stress encompassed a chronic alternating forced swim and restraint stress paradigm as described in section “Materials and Methods.”
