# Supplementary material for: Distinct Cecal and Fecal Microbiome Responses to Stress Are Accompanied by Sex- and Diet-Dependent Changes in Behavior and Gut Serotonin
Source: Front Neurosci. 2022 Apr 12;16:827343. doi: 10.3389/fnins.2022.827343 (PMC9039258; doi:10.3389/fnins.2022.827343)
Supplement: Supplementary file 1 [file Table_1.docx]

**Supplementary Table 1. Relative abundance of bacterial phyla in cecal samples from mice**

| **Phyla** | **Relative abundance**  **(%)** |
| --- | --- |
| *Firmicutes* | 70.89 |
| *Bacteroidetes* | 21.68 |
| *Proteobacteria* | 4.00 |
| *Actinobacteria* | 1.55 |
| *Deferribacteres* | 0.91 |
| *Tenericutes* | 0.88 |
| *Patescibacteria* | 0.08 |
| *Cyanobacteria* | 0.01 |

**Supplementary Table 2. The 50 most abundant bacterial ASVs in cecal samples from mice**

|  | | | | | | **NCBI BLAST** | | |
| --- | --- | --- | --- | --- | --- | --- | --- | --- |
| **ASV** | **Relative abundance**  **(%)** | **Phylum** | **Taxonomy (Silva v138)** | | **Classification** | | **Similarity (%)** | **Accession no.** |
| ASV 4 | 3.97 | *Firmicutes* | | *Erysipelotrichaceae NYU-BL-F16* | | *Ileibacterium valens strain NYU-BL-A3* | 100 | NR_156909.1 |
| ASV 1 | 3.74 | *Firmicutes* | | *Lactobacillus* | | *Lactobacillus gasseri* | 100 | OK021660.1 |
| ASV 16 | 2.50 | *Proteobacteria* | | *Desulfovibrio* | | *Desulfovibrio sp.* | 100 | MK287684.1 |
| ASV 11 | 2.31 | *Firmicutes* | | *Roseburia* | | *Roseburia sp.* | 100 | MN081694.1 |
| ASV 7 | 2.26 | *Firmicutes* | | *Faecalibaculum* | | *Faecalibaculum rodentium* | 100 | LC416469.1 |
| ASV 14 | 2.16 | *Firmicutes* | | *Lachnospiraceae NK4A136 group* | | *Lachnospiraceae bacterium A4* | 99.59 | DQ789118.1 |
| ASV 39 | 1.81 | *Firmicutes* | | *Lachnospiraceae bacterium 10-1* | | *Lachnospiraceae sp.* | 99.18 | LC333692.1 |
| ASV 5 | 1.69 | *Firmicutes* | | *Lactobacillus* | | *Limosilactobacillus reuteri* | 100 | OK138596.1 |
| ASV 6 | 1.67 | *Firmicutes* | | *Lactobacillus murinus* | | *Lactobacillus murinus* | 100 | MT585453.1 |
| ASV 2 | 1.57 | *Bacteroidetes* | | *Muribaculaceae* | | *Paramuribaculum intestinale* | 94.21 | MG970332.1 |
| ASV 3 | 1.56 | *Bacteroidetes* | | *Muribaculaceae* | | *Paramuribaculum intestinale* | 100 | MG970332.1 |
| ASV 8 | 1.55 | *Bacteroidetes* | | *Muribaculaceae* | | *Muribaculum sp.* | 95.04 | AY239469.1 |
| ASV 9 | 1.50 | *Bacteroidetes* | | *Muribaculaceae* | | *Muribaculum sp.* | 100 | MK287695.1 |
| ASV 13 | 1.43 | *Bacteroidetes* | | *Muribaculaceae* | | *Muribaculum intestinale* | 93.39 | MN904451.1 |
| ASV 23 | 1.33 | *Firmicutes* | | *Lachnospiraceae UCG-001* | | *Anaerostipes sp.* | 95.44 | MN081669.1 |
| ASV 40 | 1.25 | *Firmicutes* | | *Lachnospiraceae* | | *Kineothrix alysoides* | 97.53 | NR_156080.1 |
| ASV 12 | 1.20 | *Firmicutes* | | *Lactobacillus* | | *Lactobacillus intestinalis* | 100 | MN081712.1 |
| ASV 18 | 1.20 | *Bacteroidetes* | | *Muribaculaceae* | | *Muribaculaceae sp.* | 92.92 | MN264626.1 |
| ASV 33 | 1.19 | *Firmicutes* | | *Lachnospiraceae NK4A136 group* | | *Fusimonas intestini* | 97.53 | AB861470.1 |
| ASV 28 | 1.17 | *Firmicutes* | | *Roseburia* | | *Eubacterium plexicaudatum* | 97.12 | AF157058.1 |
| ASV 17 | 1.17 | *Firmicutes* | | *Lachnospiraceae NK4A136 group* | | *Fusimonas intestini* | 98.35 | AB861470.1 |
| ASV 10 | 1.16 | *Bacteroidetes* | | *Muribaculaceae* | | *Muribaculum sp.* | 100 | MK521455.1 |
| ASV 20 | 1.16 | *Bacteroidetes* | | *Muribaculaceae* | | *Muribaculum sp.* | 93.33 | MK287695.1 |
| ASV 22 | 1.11 | *Firmicutes* | | *Dubosiella* | | *Erysipelotrichaceae sp.* | 97.11 | MK929057.1 |
| ASV 35 | 1.09 | *Firmicutes* | | *Roseburia* | | *Eubacterium plexicaudatum* | 97.94 | AF157058.1 |
| ASV 32 | 1.05 | *Bacteroidetes* | | *Muribaculaceae* | | *Muribaculaceae sp.* | 95.04 | MN264626.1 |
| ASV 15 | 0.91 | *Deferribacteres* | | *Mucispirillum* | | *Mucispirillum schaedleri* | 100 | AY387668.1 |
| ASV 29 | 0.90 | *Firmicutes* | | *Lachnospiraceae UCG-001* | | *Anaerostipes sp* | 96.68 | MN081669.1 |
| ASV 43 | 0.87 | *Proteobacteria* | | *Desulfovibrio* | | *Desulfovibrio sp.* | 99.59 | MT065886.1 |
| ASV 45 | 0.82 | *Firmicutes* | | *Lachnospiraceae NK4A136 group* | | *Kineothrix sp.* | 95.88 | MN081711.1 |
| ASV 55 | 0.82 | *Firmicutes* | | *Lachnospiraceae NK4A136 group* | | *Kineothrix sp.* | 100 | MN081711.1 |
| ASV 52 | 0.75 | *Firmicutes* | | *Clostridium sp. Culture-41* | | *Clostridium sp.* | 98.77 | AB622820.1 |
| ASV 25 | 0.66 | *Firmicutes* | | *Allobaculum* | | *Allobaculum sp.* | 100 | MZ153115.1 |
| ASV 41 | 0.64 | *Firmicutes* | | *Anaerotruncus* | | *Anaerotruncus sp.* | 100 | MN081629.1 |
| ASV 30 | 0.60 | *Actinobacteria* | | *Bifidobacterium* | | *Bifidobacterium pseudolongum* | 100 | LC527458.1 |
| ASV 42 | 0.55 | *Bacteroidetes* | | *Muribaculaceae* | | *Bacteroidales sp.* | 86.89 | MK929084.1 |
| ASV 46 | 0.55 | *Firmicutes* | | *unidentified* | | *Clostridiales sp.* | 93.42 | JF733451.1 |
| ASV 37 | 0.54 | *Bacteroidetes* | | *Muribaculaceae* | | *Paramuribaculum intestinale* | 99.59 | MG970332.1 |
| ASV 26 | 0.54 | *Bacteroidetes* | | *Rikenellaceae RC9 gut group* | | *Ralstonia solanacearum* | 90.57 | CP011998.1 |
| ASV 19 | 0.53 | *Bacteroidetes* | | *Bacteroides* | | *Bacteroides rodentium* | 100 | MK287703.1 |
| ASV 24 | 0.53 | *Bacteroidetes* | | *Alistipes* | | *Alistipes dispar* | 97.12 | AP019736.1 |
| ASV 61 | 0.50 | *Firmicutes* | | *mouse gut metagenome* | | *Lachnospiraceae sp.* | 100 | KX009932.1 |
| ASV 27 | 0.47 | *Bacteroidetes* | | *Bacteroides* | | *Bacteroides acidifaciens* | 100 | MT560834.1 |
| ASV 75 | 0.47 | *Firmicutes* | | *Lachnospiraceae NK4A136 group* | | *Fusimonas intestini* | 97.12 | AB861470.1 |
| ASV 101 | 0.46 | *Firmicutes* | | *Staphylococcus* | | *Staphylococcus xylosus* | 100 | OK083801.1 |
| ASV 92 | 0.46 | *Firmicutes* | | *Lachnospiraceae NK4A136 group* | | *Kineothrix sp.* | 96.71 | MN081711.1 |
| ASV 82 | 0.45 | *Firmicutes* | | *Lachnospiraceae NK4A136 group* | | *Lachnospiraceae* | 97.53 | KX009918.1 |
| ASV 50 | 0.45 | *Firmicutes* | | *Lachnospiraceae NK4A136 group* | | *Kineothrix sp.* | 97.54 | MN081711.1 |
| ASV 62 | 0.44 | *Firmicutes* | | *Lachnospiraceae NK4A136 group* | | *Lachnospiraceae sp.* | 100 | KX009918.1 |
| ASV 44 | 0.44 | *Firmicutes* | | *Lachnospiraceae* | | *Lachnospiraceae sp.* | 95.47 | LC333726.1 |

**Supplementary Table 3. Significant model effects of stress, sex and diet comparing murine cecal microbiota community structure.** After correction for multiple testing using Tukey’s HSD, pairwise comparisons between the effects of levels of stress and diet on Chao species richness were no longer significant.

| Type 3 Tests of Fixed Effects on Chao Species Richness of Cecal Murine Microbial Communities | | | | | |
| --- | --- | --- | --- | --- | --- |
| Effect | Num DF | Den DF | F Value | P value | Sig |
| Sex | 1 | 69 | 1.2 | 0.278 |  |
| Stress | 1 | 69 | 0.2 | 0.659 |  |
| Diet | 1 | 69 | 2.58 | 0.113 |  |
| Sex*Stress | 1 | 69 | 0 | 0.959 |  |
| Sex*Diet | 1 | 69 | 0.13 | 0.723 |  |
| Stress*Diet | 1 | 69 | 4.12 | 0.046 | . |
| Sex*Stress*Diet | 1 | 69 | 0.59 | 0.446 |  |
|  |  |  |  |  |  |
| Type 3 Tests of Fixed Effects on Simpson Community Evenness of Cecal Murine Microbial Communities | | | | | |
| Effect | Num DF | Den DF | F Value | P value | Sig |
| Sex | 1 | 69 | 4.9 | 0.030 | . |
| Stress | 1 | 69 | 0.1 | 0.752 |  |
| Diet | 1 | 69 | 2.04 | 0.158 |  |
| Sex*Stress | 1 | 69 | 0 | 0.972 |  |
| Sex*Diet | 1 | 69 | 3.94 | 0.051 |  |
| Stress*Diet | 1 | 69 | 0.02 | 0.900 |  |
| Sex*Stress*Diet | 1 | 69 | 0.06 | 0.811 |  |
|  |  |  |  |  |  |
| Type 3 Tests of Fixed Effects on Shannon Diversity of Cecal Murine Microbial Communities | | | | | |
| Effect | Num DF | Den DF | F Value | P value | Sig |
| Sex | 1 | 69 | 6.26 | 0.015 | . |
| Stress | 1 | 69 | 0.42 | 0.519 |  |
| Diet | 1 | 69 | 0.02 | 0.877 |  |
| Sex*Stress | 1 | 69 | 0 | 0.949 |  |
| Sex*Diet | 1 | 69 | 1.99 | 0.163 |  |
| Stress*Diet | 1 | 69 | 0.16 | 0.687 |  |
| Sex*Stress*Diet | 1 | 69 | 0.23 | 0.631 |  |

* denotes interaction between the fixed effects listed; . denotes significance (P < 0.05)

**Supplementary Table S4. PERMANOVA results when using Eq. 1 to determine differences in cecal microbiota between of mice.**

| **PERMANOVA** | | | | | |  |
| --- | --- | --- | --- | --- | --- | --- |
| adonis2(formula = data_bray ~ Stress + Sex + Diet + Stress * Sex + Stress * Diet + Sex * Diet + Stress * Diet * Sex, data = data_sub_type_df) | | | | | |  |
| Permutation: free | | | | | |  |
| Number of permutations: 999 | | | | | |  |
|  | Df | SumsOfSqs | F.Model | R2 | P-value | sig |
| Stress | 1 | 0.1956 | 0.01248 | 1.0835 | 0.332 |  |
| Sex | 1 | 1.0658 | 0.06797 | 5.9034 | 0.001 | . |
| Diet | 1 | 0.9832 | 0.0627 | 5.4457 | 0.001 | . |
| Stress*Sex | 1 | 0.1685 | 0.01075 | 0.9334 | 0.558 |  |
| Stress*Diet | 1 | 0.1958 | 0.01249 | 1.0844 | 0.31 |  |
| Sex*Diet | 1 | 0.3255 | 0.02076 | 1.8028 | 0.005 | . |
| Stress*Sex*Diet | 1 | 0.2878 | 0.01836 | 1.5942 | 0.029 | . |
| Residual | 69 | 12.4575 | 0.7945 |  |  |  |
| Total | 76 | 15.6797 | 1 |  |  |  |

* denotes interaction between the fixed effects listed; . denotes significance (P < 0.05).

**Supplementary Table S5. Pairwise PERMANOVA comparisons of Bray-Curtis dissimilarity measures in mice cecal microbial communities across all levels for stress, sex and diet based on significant interaction effect seen in Supplementary Table S4 results.**

| **Pairwise PERMANOVA** | | | | | | | | | | | | | |
| --- | --- | --- | --- | --- | --- | --- | --- | --- | --- | --- | --- | --- | --- |
|  |  |  |  |  |  |  |  |  |  |  |  |  |  |
|  |  |  |  |  |  |  |  |  |  |  |  |  |  |
|  |  |  |  |  |  |  |  |  |  |  |  |  |  |
| 1^st^ group | | |  | 2^nd^ group | | | Df | SumsOfSqs | F.Model | R2 | P value | Q value | sig |
| F | B | S | VS | F | B | NS | 1 | 0.275 | 1.586 | 0.081 | 0.033 | 0.924 |  |
| F | B | S | VS | F | SC | S | 1 | 0.513 | 2.941 | 0.140 | 0.001 | 0.028 | . |
| F | B | S | VS | F | SC | NS | 1 | 0.514 | 2.864 | 0.137 | 0.001 | 0.028 | . |
| F | B | NS | VS | F | SC | S | 1 | 0.393 | 2.221 | 0.110 | 0.003 | 0.084 |  |
| F | B | NS | VS | F | SC | NS | 1 | 0.467 | 2.568 | 0.125 | 0.001 | 0.028 | . |
| F | SC | S | VS | F | SC | NS | 1 | 0.201 | 1.102 | 0.058 | 0.307 | 1 |  |
| F | B | S | VS | M | B | S | 1 | 0.513 | 3.083 | 0.154 | 0.001 | 0.028 | . |
| F | B | NS | VS | M | B | NS | 1 | 0.398 | 2.109 | 0.116 | 0.003 | 0.084 |  |
| F | SC | S | VS | M | SC | S | 1 | 0.579 | 3.214 | 0.152 | 0.001 | 0.028 | . |
| F | SC | NS | VS | M | SC | NS | 1 | 0.350 | 1.873 | 0.094 | 0.003 | 0.084 |  |
| F | SC | NS | VS | M | SC | S | 1 | 0.474 | 2.563 | 0.125 | 0.001 | 0.028 | . |
| F | SC | S | VS | M | SC | NS | 1 | 0.379 | 2.082 | 0.104 | 0.002 | 0.056 |  |
| F | B | NS | VS | M | B | S | 1 | 0.374 | 2.217 | 0.115 | 0.004 | 0.112 |  |
| F | B | S | VS | M | B | NS | 1 | 0.530 | 2.848 | 0.151 | 0.001 | 0.028 | . |
| F | B | S | VS | M | SC | S | 1 | 0.863 | 4.879 | 0.213 | 0.001 | 0.028 | . |
| F | B | NS | VS | M | SC | NS | 1 | 0.473 | 2.608 | 0.127 | 0.001 | 0.028 | . |
| F | SC | S | VS | M | B | S | 1 | 0.547 | 3.222 | 0.159 | 0.001 | 0.028 | . |
| F | SC | NS | VS | M | B | NS | 1 | 0.524 | 2.683 | 0.144 | 0.001 | 0.028 | . |
| F | B | S | VS | M | SC | NS | 1 | 0.727 | 4.067 | 0.184 | 0.001 | 0.028 | . |
| F | B | NS | VS | M | SC | S | 1 | 0.708 | 3.946 | 0.180 | 0.001 | 0.028 | . |
| F | SC | S | VS | M | B | NS | 1 | 0.506 | 2.665 | 0.143 | 0.001 | 0.028 | . |
| F | SC | NS | VS | M | B | S | 1 | 0.589 | 3.368 | 0.165 | 0.001 | 0.028 | . |
| M | B | S | VS | M | B | NS | 1 | 0.167 | 0.920 | 0.058 | 0.603 | 1 |  |
| M | B | S | VS | M | SC | S | 1 | 0.459 | 2.664 | 0.135 | 0.002 | 0.056 |  |
| M | B | S | VS | M | SC | NS | 1 | 0.348 | 1.997 | 0.105 | 0.005 | 0.14 |  |
| M | B | NS | VS | M | SC | S | 1 | 0.412 | 2.138 | 0.118 | 0.006 | 0.168 |  |
| M | B | NS | VS | M | SC | NS | 1 | 0.356 | 1.825 | 0.102 | 0.029 | 0.812 |  |
| M | SC | S | VS | M | SC | NS | 1 | 0.205 | 1.113 | 0.058 | 0.276 | 1 |  |

F or M denotes female or male mice groups; SC or B denotes chow diet or beef-chow mix diet groups; S or NS denotes stress or no stress groups; . denotes significance (Q < 0.05)

**Supplementary Table S6. Differences in ASVs between cecal microbiota of male mice compared to female mice.** ASVs are significantly different based on fixed effect of sex.

| **ASV** | **Taxonomy (Silva v138)** | **Comparison** | **More abundant in** | **Log2FC** | **Q value** |
| --- | --- | --- | --- | --- | --- |
| ASV16 | *Desulfovibrio* | M vs. F | F | 0.74 | 0.002 |
| ASV52 | *Clostridium sp. Culture-41* | M vs. F | F | 0.69 | 0.008 |
| ASV30 | *Bifidobacterium* | M vs. F | M | 1.09 | 0.019 |
| ASV46 | *Ruminococcaceae UCG-014* | M vs. F | M | 1.92 | 0.027 |

F or M denotes female or male mice groups

**Supplementary Table S7. Differences in ASVs between cecal microbiota of mice fed the standard chow diet compared those fed the beef supplemented diet.** ASVs are significantly different based on fixed effect of diet.

| **ASV** | **Taxonomy (Silva v138)** | **Comparison** | **More abundant in** | **Log2FC** | **Q value** |
| --- | --- | --- | --- | --- | --- |
| ASV6 | *Lactobacillus murinus* | B vs. SC | B | 0.65 | 0.007 |
| ASV58 | *Lactobacillus murinus* | B vs. SC | B | 3.17 | <0.0001 |

SC or B denotes chow diet or beef-chow mix diet groups.

**Supplementary Table 8. Relative abundance of bacterial phyla in fecal samples from mice**

| **Phyla** | **Relative abundance**  **(%)** |
| --- | --- |
| *Firmicutes* | 54.97 |
| *Bacteroidetes* | 38.24 |
| *Actinobacteria* | 2.60 |
| *Proteobacteria* | 2.15 |
| *Deferribacteres* | 1.05 |
| *Tenericutes* | 0.90 |
| *Patescibacteria* | 0.09 |
| *Cyanobacteria* | 0.01 |

**Supplementary Table 9. The 50 most abundant bacterial ASVs in fecal samples from mice**

|  | | | | **NCBI BLAST** | | | |
| --- | --- | --- | --- | --- | --- | --- | --- |
| **ASV** | **Relative abundance**  **(%)** | **Phylum** | **Taxonomy (Silva v138)** | | **Classification** | **Similarity (%)** | **Accession no.** |
| ASV 1 | 5.84 | *Firmicutes* | *Lactobacillus* | *Lactobacillus gasseri* | | 100 | OK021660.1 |
| ASV 4 | 4.97 | *Firmicutes* | *Erysipelotrichaceae NYU-BL-F16* | *Ileibacterium valens strain NYU-BL-A3* | | 100 | NR_156909.1 |
| ASV 7 | 4.09 | *Firmicutes* | *Faecalibaculum* | *Faecalibaculum rodentium* | | 100 | LC416469.1 |
| ASV 3 | 3.41 | *Bacteroidetes* | *Muribaculaceae* | *Paramuribaculum intestinale* | | 100 | MG970332.1 |
| ASV 2 | 3.39 | *Bacteroidetes* | *Muribaculaceae* | *Paramuribaculum intestinale* | | 94.21 | MG970332.1 |
| ASV 6 | 3.14 | *Firmicutes* | *Lactobacillus murinus* | *Lactobacillus murinus* | | 100 | MT585453.1 |
| ASV 8 | 2.86 | *Bacteroidetes* | *Muribaculaceae* | *Muribaculum sp.* | | 95.04 | AY239469.1 |
| ASV 5 | 2.83 | *Firmicutes* | *Lactobacillus* | *Limosilactobacillus reuteri* | | 100 | OK138596.1 |
| ASV 10 | 2.76 | *Bacteroidetes* | *Muribaculaceae* | *Muribaculum sp.* | | 100 | MK521455.1 |
| ASV 9 | 2.40 | *Bacteroidetes* | *Muribaculaceae* | *Muribaculum sp.* | | 100 | MK287695.1 |
| ASV 12 | 2.09 | *Firmicutes* | *Lactobacillus* | *Lactobacillus intestinalis* | | 100 | MN081712.1 |
| ASV 13 | 2.01 | *Bacteroidetes* | *Muribaculaceae* | *Muribaculum intestinale* | | 93.39 | MN904451.1 |
| ASV 18 | 1.92 | *Bacteroidetes* | *Muribaculaceae* | *Muribaculaceae sp.* | | 92.92 | MN264626.1 |
| ASV 20 | 1.86 | *Bacteroidetes* | *Muribaculaceae* | *Muribaculum sp.* | | 93.33 | MK287695.1 |
| ASV 22 | 1.71 | *Firmicutes* | *Dubosiella* | *Erysipelotrichaceae sp.* | | 97.11 | MK929057.1 |
| ASV 32 | 1.50 | *Bacteroidetes* | *Muribaculaceae* | *Muribaculaceae sp.* | | 95.04 | MN264626.1 |
| ASV 30 | 1.38 | *Actinobacteria* | *Bifidobacterium* | *Bifidobacterium pseudolongum* | | 100 | LC527458.1 |
| ASV 26 | 1.31 | *Bacteroidetes* | *Rikenellaceae RC9 gut group* | *Ralstonia solanacearum* | | 90.57 | CP011998.1 |
| ASV 25 | 1.19 | *Firmicutes* | *Allobaculum* | *Allobaculum sp.* | | 100 | MZ153115.1 |
| ASV 37 | 1.19 | *Bacteroidetes* | *Muribaculaceae* | *Paramuribaculum intestinale* | | 99.59 | MG970332.1 |
| ASV 16 | 1.10 | *Proteobacteria* | *Desulfovibrio* | *Desulfovibrio sp.* | | 100 | MK287684.1 |
| ASV 14 | 1.09 | *Firmicutes* | *Lachnospiraceae NK4A136 group* | *Lachnospiraceae bacterium A4* | | 99.59 | DQ789118.1 |
| ASV 11 | 1.07 | *Firmicutes* | *Roseburia* | *Roseburia sp.* | | 100 | MN081694.1 |
| ASV 15 | 1.03 | *Deferribacteres* | *Mucispirillum* | *Mucispirillum schaedleri* | | 100 | AY387668.1 |
| ASV 36 | 0.94 | *Bacteroidetes* | *Muribaculaceae* | *Muribaculum sp.* | | 92.56 | MK287701.1 |
| ASV 46 | 0.88 | *Firmicutes* | *unidentified Clostridiales* | *Clostridiales sp.* | | 93.42 | JF733451.1 |
| ASV 19 | 0.85 | *Bacteroidetes* | *Bacteroides* | *Bacteroides rodentium* | | 100 | MK287703.1 |
| ASV 101 | 0.74 | *Firmicutes* | *Staphylococcus* | *Staphylococcus xylosus* | | 100 | OK083801.1 |
| ASV 27 | 0.72 | *Bacteroidetes* | *Bacteroides* | *Bacteroides acidifaciens* | | 100 | MT560834.1 |
| ASV 23 | 0.69 | *Firmicutes* | *Lachnospiraceae UCG-001* | *Anaerostipes sp.* | | 95.44 | MN081669.1 |
| ASV 77 | 0.67 | *Firmicutes* | *[Eubacterium] ventriosum group* | *Eubacterium ventriosum* | | 97.12 | LC515582.1 |
| ASV 58 | 0.67 | *Firmicutes* | *Lactobacillus murinus* | *Lactobacillus murinus* | | 99.59 | MT585453.1 |
| ASV 42 | 0.66 | *Bacteroidetes* | *Muribaculaceae* | *Bacteroidales sp.* | | 86.89 | MK929084.1 |
| ASV 51 | 0.64 | *Bacteroidetes* | *Rikenellaceae RC9 gut group* | *Ralstonia solanacearum* | | 90.16 | CP011998.1 |
| ASV 39 | 0.63 | *Firmicutes* | *Lachnospiraceae bacterium 10-1* | *Lachnospiraceae sp.* | | 99.18 | LC333692.1 |
| ASV 69 | 0.61 | *Bacteroidetes* | *Muribaculaceae* | *Bacteroidales sp.* | | 92.15 | MK287702.1 |
| ASV 48 | 0.57 | *Bacteroidetes* | *Muribaculaceae* | *Muribaculaceae sp.* | | 90.95 | MN264626.1 |
| ASV 38 | 0.52 | *Bacteroidetes* | *Muribaculaceae* | *Duncaniella dubosii* | | 100 | MK521458.1 |
| ASV 33 | 0.52 | *Firmicutes* | *Lachnospiraceae NK4A136 group* | *Fusimonas intestini* | | 97.53 | AB861470.1 |
| ASV 17 | 0.50 | *Firmicutes* | *Lachnospiraceae NK4A136 group* | *Fusimonas intestini* | | 98.35 | AB861470.1 |
| ASV 49 | 0.49 | *Bacteroidetes* | *Muribaculaceae* | *Muribaculum sp.* | | 100 | MK287697.1 |
| ASV 24 | 0.45 | *Bacteroidetes* | *Alistipes* | *Alistipes dispar* | | 97.12 | AP019736.1 |
| ASV 99 | 0.44 | *Firmicutes* | *Lactobacillus murinus* | *Lactobacillus murinus* | | 99.59 | MT585453.1 |
| ASV 56 | 0.41 | *Bacteroidetes* | *Muribaculaceae* | *Muribaculum sp.* | | 99.59 | MN081656.1 |
| ASV 29 | 0.41 | *Firmicutes* | *Lachnospiraceae UCG-001* | *Anaerostipes sp.* | | 96.68 | MN081669.1 |
| ASV 65 | 0.40 | *Bacteroidetes* | *Alistipes* | *Alistipes shahii* | | 95.88 | MN537530.1 |
| ASV 40 | 0.39 | *Firmicutes* | *Lachnospiraceae* | *Kineothrix alysoides* | | 97.53 | NR_156080.1 |
| ASV 83 | 0.37 | *Proteobacteria* | *Parasutterella* | *Parasutterella sp.* | | 100 | MN904456.1 |
| ASV 59 | 0.37 | *Bacteroidetes* | *Muribaculaceae* | *Bacteroidales bacterium* | | 93.42 | MK929084.1 |
| ASV 54 | 0.34 | *Bacteroidetes* | *Alistipes* | *Alistipes finegoldii* | | 96.71 | NR_102944.1 |

**Supplementary Table S10. Significant model effects of stress, sex and diet comparing murine fecal microbiota community structure.**

| Type 3 Tests of Fixed Effects on Chao Species Richness of Fecal Murine Microbial Communities | | | | | |
| --- | --- | --- | --- | --- | --- |
| Effect | Num DF | Den DF | F Value | P value | Sig |
| Sex | 1 | 68 | 12.62 | 0.001 | . |
| Stress | 1 | 68 | 1.96 | 0.167 |  |
| Diet | 1 | 68 | 4.86 | 0.031 | . |
| Sex*Stress | 1 | 68 | 6.3 | 0.014 | . |
| Sex*Diet | 1 | 68 | 1.74 | 0.191 |  |
| Stress*Diet | 1 | 68 | 0.29 | 0.594 |  |
| Sex*Stress*Diet | 1 | 68 | 3.19 | 0.079 |  |
|  |  |  |  |  |  |
| Type 3 Tests of Fixed Effects on Simpson Community Evenness of Fecal Murine Microbial Communities | | | | | |
| Effect | Num DF | Den DF | F Value | P value | Sig |
| Sex | 1 | 68 | 0.02 | 0.885 |  |
| Stress | 1 | 68 | 2.92 | 0.092 |  |
| Diet | 1 | 68 | 0.81 | 0.371 |  |
| Sex*Stress | 1 | 68 | 2.7 | 0.105 |  |
| Sex*Diet | 1 | 68 | 10.24 | 0.002 | . |
| Stress*Diet | 1 | 68 | 0.31 | 0.580 |  |
| Sex*Stress*Diet | 1 | 68 | 4.92 | 0.030 | . |
|  |  |  |  |  |  |
| Type 3 Tests of Fixed Effects on Shannon Diversity of Fecal Murine Microbial Communities | | | | | |
| Effect | Num DF | Den DF | F Value | P value | Sig |
| Sex | 1 | 68 | 2.92 | 0.092 |  |
| Stress | 1 | 68 | 4.68 | 0.034 | . |
| Diet | 1 | 68 | 0.58 | 0.449 |  |
| Sex*Stress | 1 | 68 | 3.9 | 0.052 |  |
| Sex*Diet | 1 | 68 | 8.03 | 0.006 | . |
| Stress*Diet | 1 | 68 | 0 | 0.986 |  |
| Sex*Stress*Diet | 1 | 68 | 7.46 | 0.008 | . |

* denotes interaction between the fixed effects listed; . denotes significance (P < 0.05)

**Supplementary Table S11. Pairwise comparisons across levels of stress, sex and diet when significant interaction effects affected murine fecal microbiota community structure.** Based on values presented in Supplementary Table S6.

| **Interaction Effect** | **1st group** | | |  | **2nd group** | | | **Q value** | **Sig** |
| --- | --- | --- | --- | --- | --- | --- | --- | --- | --- |
|  |  | | |  |  | | |  |  |
| **Chao Species Richness** | | | | | | | | |  |
| Sex*Stress | F | NS |  | vs | M | S |  | 0.005 | . |
| Sex*Stress | F | S |  | vs | M | S |  | <0.001 | . |
|  |  |  |  |  |  |  |  |  |  |
| **Simpson Community Evenness** | | | | | | | | |  |
| Sex*Stress*Diet | F | NS | SC | vs | M | NS | SC | 0.013 | . |
| Sex*Stress*Diet | M | NS | SC | vs | M | S | B | 0.009 | . |
| Sex*Stress*Diet | M | NS | SC | vs | M | S | SC | 0.048 | . |
|  |  |  |  |  |  |  |  |  |  |
| **Shannon Species Diversity** | | | | | | | | |  |
| Sex*Stress*Diet | F | NS | B | vs | M | S | B | 0.035 | . |
| Sex*Stress*Diet | F | NS | B | vs | M | S | SC | 0.010 | . |
| Sex*Stress*Diet | M | NS | SC | vs | M | S | SC | 0.016 | . |

* denotes interaction between the fixed effects listed; F or M denotes female or male mice groups; SC or B denotes chow diet or beef-chow mix diet groups when applicable; S or NS denotes stress or no stress groups; . denotes significance (Q < 0.05).

**Supplementary Table 12. PERMANOVA results when using Eq. 1 to determine differences in fecal microbiota between of mice.**

| **PERMANOVA** | | | | | |  |
| --- | --- | --- | --- | --- | --- | --- |
| adonis2(formula = data_bray ~ Stress + Sex + Diet + Stress * Sex + Stress * Diet + Sex * Diet + Stress * Diet * Sex, data = data_sub_type_df) | | | | | |  |
| Permutation: free | | | | | |  |
| Number of permutations: 999 | | | | | |  |
|  | Df | SumsOfSqs | F.Model | R2 | P value | sig |
| Stress | 1 | 0.2968 | 0.02058 | 1.8776 | 0.024 | . |
| Sex | 1 | 1.0998 | 0.07626 | 6.9577 | 0.001 | . |
| Diet | 1 | 0.9249 | 0.06412 | 5.8507 | 0.001 | . |
| Stress*Sex | 1 | 0.1834 | 0.01271 | 1.1601 | 0.266 |  |
| Stress*Diet | 1 | 0.2156 | 0.01495 | 1.3639 | 0.133 |  |
| Sex*Diet | 1 | 0.5743 | 0.03982 | 3.6332 | 0.001 | . |
| Stress*Sex*Diet | 1 | 0.3788 | 0.02626 | 2.3963 | 0.003 | . |
| Residual | 68 | 10.7491 | 0.74529 |  |  |  |
| Total | 75 | 14.4228 | 1 |  |  |  |

* denotes interaction between the fixed effects listed; . denotes significance (P < 0.05).

**Supplementary Table 13. Pairwise PERMANOVA comparisons of Bray-Curtis dissimilarity measures in mice fecal microbial communities across all levels for Stress, Sex and Diet based on significant interaction effect seen in Supplementary Table S12 results.**

| **Pairwise PERMANOVA** | | | | | | | | | | | | | |
| --- | --- | --- | --- | --- | --- | --- | --- | --- | --- | --- | --- | --- | --- |
|  |  |  |  |  |  |  |  |  |  |  |  |  |  |
|  |  |  |  |  |  |  |  |  |  |  |  |  |  |
|  |  |  |  |  |  |  |  |  |  |  |  |  |  |
| 1^st^ group | | |  | 2^nd^ group | | | Df | SumsOfSqs | F.Model | R2 | P value | Q value | sig |
| F | B | S | VS | F | B | NS | 1 | 0.38 | 2.88 | 0.15 | 0.006 | 0.168 |  |
| F | B | S | VS | F | SC | S | 1 | 0.80 | 5.57 | 0.24 | 0.001 | 0.028 | . |
| F | B | S | VS | F | CS | NS | 1 | 0.74 | 5.27 | 0.24 | 0.001 | 0.028 | . |
| F | B | NS | VS | F | CS | S | 1 | 0.47 | 3.17 | 0.16 | 0.002 | 0.056 |  |
| F | B | NS | VS | F | CS | NS | 1 | 0.50 | 3.41 | 0.18 | 0.001 | 0.028 | . |
| F | SC | S | VS | F | CS | NS | 1 | 0.13 | 0.81 | 0.05 | 0.679 | 1 |  |
| F | B | S | VS | M | B | S | 1 | 0.54 | 3.91 | 0.19 | 0.001 | 0.028 | . |
| F | B | NS | VS | M | B | NS | 1 | 0.62 | 3.47 | 0.18 | 0.001 | 0.028 | . |
| F | SC | S | VS | M | CS | S | 1 | 0.71 | 4.47 | 0.20 | 0.001 | 0.028 | . |
| F | SC | NS | VS | M | CS | NS | 1 | 0.35 | 2.20 | 0.11 | 0.015 | 0.42 |  |
| F | SC | NS | VS | M | CS | S | 1 | 0.50 | 3.21 | 0.16 | 0.002 | 0.056 |  |
| F | SC | S | VS | M | CS | NS | 1 | 0.38 | 2.40 | 0.12 | 0.017 | 0.476 |  |
| F | B | NS | VS | M | B | S | 1 | 0.47 | 3.28 | 0.17 | 0.001 | 0.028 | . |
| F | B | S | VS | M | B | NS | 1 | 0.79 | 4.66 | 0.22 | 0.001 | 0.028 | . |
| F | B | S | VS | M | CS | S | 1 | 1.04 | 7.32 | 0.29 | 0.001 | 0.028 | . |
| F | B | NS | VS | M | CS | NS | 1 | 0.52 | 3.48 | 0.17 | 0.003 | 0.084 |  |
| F | SC | S | VS | M | B | S | 1 | 0.48 | 3.07 | 0.15 | 0.002 | 0.056 |  |
| F | SC | NS | VS | M | B | NS | 1 | 0.38 | 2.03 | 0.11 | 0.032 | 0.896 |  |
| F | B | S | VS | M | CS | NS | 1 | 0.91 | 6.32 | 0.26 | 0.001 | 0.028 | . |
| F | B | NS | VS | M | CS | S | 1 | 0.93 | 6.29 | 0.27 | 0.001 | 0.028 | . |
| F | SC | S | VS | M | B | NS | 1 | 0.50 | 2.68 | 0.14 | 0.004 | 0.112 |  |
| F | SC | NS | VS | M | B | S | 1 | 0.35 | 2.29 | 0.13 | 0.003 | 0.084 |  |
| M | B | S | VS | M | B | NS | 1 | 0.24 | 1.27 | 0.07 | 0.189 | 1 |  |
| M | B | S | VS | M | CS | S | 1 | 0.43 | 2.75 | 0.14 | 0.001 | 0.028 | . |
| M | B | S | VS | M | CS | NS | 1 | 0.32 | 2.05 | 0.11 | 0.029 | 0.812 |  |
| M | B | NS | VS | M | CS | S | 1 | 0.43 | 2.30 | 0.12 | 0.004 | 0.112 |  |
| M | B | NS | VS | M | CS | NS | 1 | 0.37 | 1.97 | 0.10 | 0.038 | 1 |  |
| M | SC | S | VS | M | CS | NS | 1 | 0.32 | 2.04 | 0.10 | 0.035 | 0.98 |  |

F or M denotes female or male mice groups; SC or B denotes chow diet or beef-chow mix diet groups; S or NS denotes stress or no stress groups; . denotes significance (Q < 0.05).

**Supplementary Table 14. Differences in ASVs between fecal microbiota of male mice compared to female mice.** ASVs are significantly different based on fixed effect of sex.

| **ASV** | **Taxonomy (Silva v138)** | **Comparison** | **More abundant in** | **Log2FC** | **Q value** |
| --- | --- | --- | --- | --- | --- |
| ASV1 | *Lactobacillus* | M vs. F | F | 0.57 | 0.0035 |
| ASV2 | *Muribaculaceae* | M vs. F | F | 0.45 | 0.0255 |
| ASV6 | *Lactobacillus murinus* | M vs. F | F | 0.70 | 0.0003 |
| ASV8 | *Muribaculaceae* | M vs. F | F | 0.47 | 0.0264 |
| ASV5 | *Lactobacillus* | M vs. F | F | 0.63 | 0.0008 |
| ASV37 | *Muribaculaceae* | M vs. F | F | 0.77 | 0.0096 |
| ASV58 | *Lactobacillus murinus* | M vs. F | F | 1.17 | 0.0003 |
| ASV24 | *Alistipes* | M vs. F | F | 0.89 | 0.0003 |
| ASV45 | *Lachnospiraceae NK4A136 group* | M vs. F | F | 0.78 | 0.0062 |
| ASV91 | *Muribaculum* | M vs. F | M | 1.20 | 0.0153 |
| ASV64 | *Ruminiclostridium 9* | M vs. F | F | 0.57 | 0.0405 |
| ASV52 | *Clostridium sp. Culture-41* | M vs. F | F | 0.87 | 0.0011 |
| ASV193 | *Enterorhabdus* | M vs. F | F | 1.03 | 0.0003 |

F or M denotes female or male mice groups.

**Supplementary Table 15. Differences in ASVs between fecal microbiota of mice fed the standard chow diet compared those fed the beef supplemented diet.** ASVs are significantly different based on fixed effect of diet.

| **ASV** | **Taxonomy (Silva v138)** | **Comparison** | **More abundant in** | **Log2FC** | **Q value** |
| --- | --- | --- | --- | --- | --- |
| ASV6 | *Lactobacillus murinus* | B vs. SC | B | 0.53 | 0.008 |
| ASV12 | *Lactobacillus* | B vs. SC | SC | 0.53 | 0.045 |
| ASV101 | *Staphylococcus* | B vs. SC | B | 4.45 | 0.000 |
| ASV27 | *Bacteroides* | B vs. SC | B | 0.56 | 0.026 |
| ASV58 | *Lactobacillus murinus* | B vs. SC | B | 1.42 | <0.0001 |
| ASV83 | *Parasutterella* | B vs. SC | B | 0.70 | 0.008 |
| ASV114 | *Muribaculaceae* | B vs. SC | SC | 1.04 | 0.046 |

SC or B denotes chow diet or beef-chow mix diet group.

**Supplementary Table 16. Differences in ASVs between mice fecal microbiota significantly affected by interactions between fixed effects of Sex and Diet.**

| **OTU** | **Taxonomy (Silva v138)** | **Comparison** | **More abundant in** | **Log2FC** | **Q value** |
| --- | --- | --- | --- | --- | --- |
| ASV30 | *Bifidobacterium* | F, B vs F, SC | F, SC | 0.86 | 0.0001 |
| ASV30 | *Bifidobacterium* | F, SC vs M, SC | F, SC | 0.50 | 0.0039 |
| ASV30 | *Bifidobacterium* | F, B vs M, B | M, B | 0.51 | 0.0233 |
| ASV146 | *Muribaculaceae* | F, SC vs M, SC | M, SC | 0.51 | 0.0324 |

F or M denotes female or male mice groups; SC or B denotes chow diet or beef-chow mix diet group.
