# Supplementary figures and images for: Distinct Cecal and Fecal Microbiome Responses to Stress Are Accompanied by Sex- and Diet-Dependent Changes in Behavior and Gut Serotonin
Source: Front Neurosci. 2022 Apr 12;16:827343. doi: 10.3389/fnins.2022.827343 (PMC9039258; doi:10.3389/fnins.2022.827343)

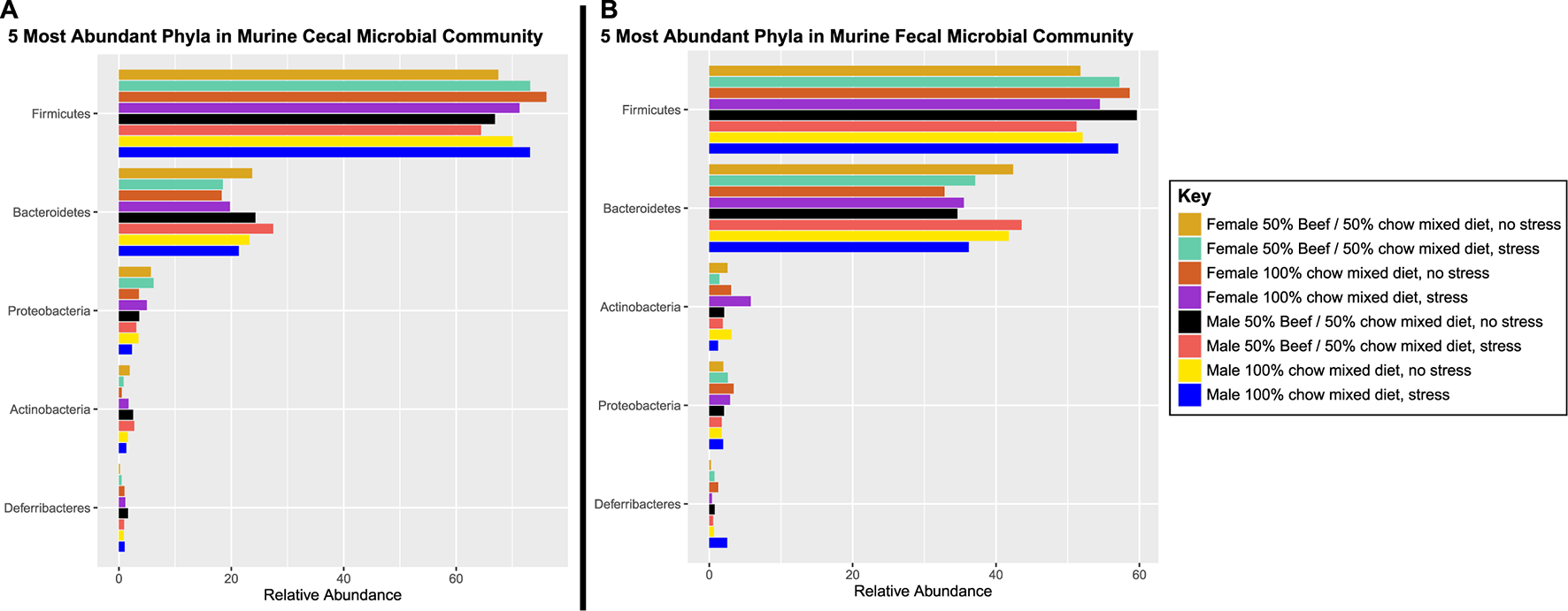

Supplement: Supplementary Figure 1 — Relative abundance of 5 most abundant phylum for cecal (A) and fecal (B) murine bacterial communities, visualized for every combination of the fixed effects diet, sex and stress. Stress encompassed a chronic alternating forced swim and restraint stress paradigm as described in section “Materials and Methods.” [file Image_1.TIF]
